# Complete chloroplast genome sequence of *Homalium hainanense* (Salicaceae)

**DOI:** 10.1080/23802359.2020.1789514

**Published:** 2020-07-15

**Authors:** Xiaojin Liu, Daping Xu, Ningnan Zhang, Zhou Hong

**Affiliations:** Research Institute of Tropical Forestry, Chinese Academy of Forestry, Guangzhou, P.R. China

**Keywords:** Chloroplast genome, *Homalium hainanense*, phylogenetic analysis

## Abstract

*Homalium hainanense* is a large evergreen tree species belonging to Salicaceae family, and its wood is tough, fine-grained, which makes it a good source of commercial use for building construction and furniture manufacturing. In this study, we sequenced the complete chloroplast genome of *H. hainanense* based on next generation sequencing and used these data to assess genomic resources. The size of the *H. hainanense* chloroplast genome was 157,852 bp, including a large single-copy region (85,888 bp), a small single-copy region (16,592 bp), and a pair of inverted repeats regions (27,686 bp). The overall GC content of the *H. hainanense* chloroplast genome was 36.6%. The plastome of *H. hainanense* was predicted to contain 112 unique genes, including 78 protein coding genes, 30 tRNA genes and 4 rRNA genes. The reconstructed phylogeny revealed that *Homalium* was monophyletic and *H. hainanense* was sister to *H. stenophyllum*, *H. paniculiflorum* and *H. racemosum.*

*Homalium hainanense*, a large tree which grows up to 30 meters tall, is a valuable tropical timber species belonging to Salicaceae family, and it was cultivated as an ornamental tree in southern China due to its excellent crown shape. In recent decades, deforestation has led to a dramatic decline in the population of *H. hainanense.* In this study, we sequenced and analyzed the chloroplast genome of *H. hainanense* based on the next-generation sequencing method (Dong et al. 2018). The objectives of this study were to establish and characterize the organization of the complete chloroplast genome of *H. hainanense* and retrieve valuable genomic resources for this species.

Fresh young leaves of *H. hainanense* were collected from a tree in Ledong, Hainan island (108°54′41″E, 18°41′02″N). The voucher specimen (Specimen accession number: RITF-LXJ2019) was deposited at the herbarium of Research Institute of Tropical Forestry, Chinese Academy of Forestry. Total genomic DNA was extracted using mCTAB protocol (Li et al. 2013). The DNA from silica dried tissue was fragmented to construct 350 bp insert library following the manufacturer’s manual (Illumina Inc., San Diego, CA, USA). The library was sequenced on the Illumina HiSeq X-ten platform at Novogene. Approximately 4 Gb data were generated from the sequencing library. The chloroplast genome was assembled with GetOrganelle (Jin et al. 2019). Plastomes were annotated with Plann (Huang and Cronk 2015). The sequence of *H. hainanense* complete chloroplast genome was submitted to GenBank, assigned with the accession number of MT478108.

The complete chloroplast genome of *H. hainanense* is 157,852 bp in length, and possesses the typical quadripartite structure including a LSC with the length of 85,888 bp separated from the 16,592 bp long SSC region by a pair of inverted repeats (IRs), each 27,686 bp. The plastome of *H. hainanense* was predicted to contain 112 unique genes, including 78 protein coding genes, 30 tRNA genes and 4 rRNA genes, respectively. Seventeen genes contain introns: 15 of them exhibit one intron and two of them contain two introns (*clpP*, and *ycf3*).

To estimate phylogenetic relationships of *H. hainanense* with other Salicaceae species. Phylogenetic analysis was conducted using published chloroplast genomes, including 27 species from Salicaceae. The chloroplast genome sequences were aligned using MAFFT v7 (Katoh and Standley 2013). Ambiguous alignment regions were trimmed by Gblocks 0.91 b (Castresana 2002). The maximum likelihood (ML) analyses were performed in RAxML v.8.1.24 (Stamatakis 2014) with branch support assessed by fast bootstrap using non-parametric bootstrap and 1000 ML pseudo-replicates. The reconstructed phylogeny revealed that *Homalium* was monophyletic and *H. hainanense* was sister to *H. stenophyllum*, *H. paniculiflorum* and *H. racemosum* (Figure 1). The genome information reported here will be useful for marker development, species discrimination, and the inference of phylogenetic relationships in the genus *Homalium.*

**Figure 1.**
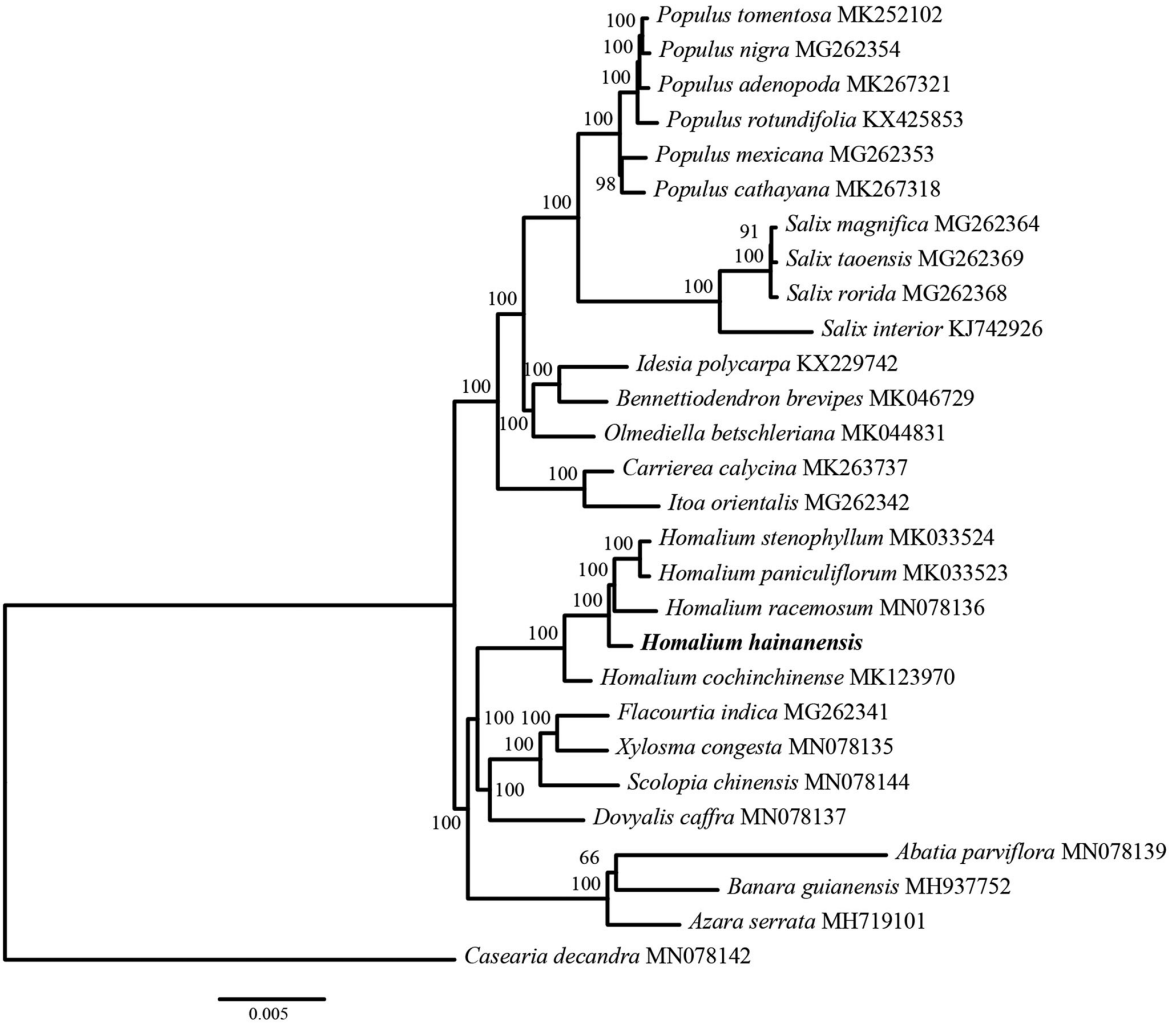
Maximum likelihood tree of Salicaceae based on the complete chloroplast genome sequences. Bootstrap support values >50% are given at the nodes.

## Data Availability

The chloroplast genome sequence of the *H. hainanense* was submitted to GenBank of NCBI (https://www.ncbi.nlm.nih.gov). The accession number from GenBank is MT478108.
